# INDIVIDUAL INTERVENTIONS TO REDUCE NIGHTTIME LIGHT EXPOSURE IN A REPRESENTATIVE SAMPLE OF ADULTS AGED 18–64 YEARS IN POLAND

**DOI:** 10.13075/ijomeh.1896.02668

**Published:** 2025

**Authors:** Radosław Sierpiński, Mateusz Jankowski, Filip Raciborski

**Affiliations:** 1 Cardinal Stefan Wyszynski University, Faculty of Medicine, Collegium Medicum, Warsaw, Poland; 2 Centre of Postgraduate Medical Education, Department of Population Health, School of Public Health, Warsaw, Poland; 3 Medical University of Warsaw, Department of Prevention of Environmental Hazards, Allergology and Immunology, Faculty of Health Sciences, Warsaw, Poland

**Keywords:** prevention, exposure, environmental health, public health, preventive measures, light pollution

## Abstract

**Objectives:**

Exposure to nighttime light pollution has a multidimensional environmental impact and is associated with harmful health effects. This study aimed to characterize individual interventions to reduce nighttime light exposure among adults in Poland and to identify factors associated with the implementation of individual interventions to reduce nighttime light exposure.

**Material and Methods:**

This is a secondary analysis of data obtained from the nationwide cross-sectional survey conducted in December 2024 in a representative sample of 5006 adults (18–64 years) in Poland. Respondents were asked about the implementation of 5 different interventions to reduce nighttime light exposure.

**Results:**

Among all respondents (N = 5006), the most common intervention to reduce nighttime light exposure was the use of blackout curtains in the bedroom (41.4% of respondents), followed by limiting screen time in the evening (17.6% of respondents). Among the respondents, 14.7% declared turning off indicator lights on electronic devices or using light bulbs with a warmer color. The least common method was using an eye mask (3.9% of respondents). In total, 65% of respondents had implemented ≥1 individual intervention to reduce exposure to nighttime light pollution. Age 18–44 years, having a full-time or part-time job, good financial situation, having sleep problems, and looking for health-related information were significantly associated (p < 0.05) with the implementation of ≥1 intervention to reduce exposure to nighttime light pollution. The most important factor associated with the intervention to reduce exposure to nighttime light pollution was active seeking health information (OR = 2.17, 95% CI: 1.9–2.48, p < 0.001). There was no significant impact of gender, educational level or place of residence (p > 0.05).

**Conclusions:**

This study revealed a gap in the implementation of individual interventions to reduce exposure to nighttime light pollution among adults in Poland. There is an urgent need to promote the prevention of environmental hazards like light pollution among adults in Poland.

## Highlights

- Personal interventions to reduce exposure to nighttime light pollution were analyzed.
- The use of blackout curtains in the bedroom was the most common intervention.
- Overall 65.0% of adults aged 18–64 years had implemented ≥1 intervention.

## INTRODUCTION

Nighttime light pollution can be defined as excessive, misdirected, or obtrusive use of artificial light during nighttime hours, that alters natural patterns of light and darkness in the environment [1–3]. The most common sources of nighttime light pollution are: streetlights, billboards, residential lighting, industrial facilities, and vehicle headlights [1,2]. Moreover, light pollution can be caused by blue light emitted by the display screens (e.g., smartphones, TV screens, tablets, computer screens, and other mobile devices) [4]. Nighttime light pollution is a growing environmental issue that results from intensified urbanization and industrialization [2,5]. It is estimated that even 80% of the global population can be exposed to light pollution [2,5].

Exposure to nighttime light pollution has a multidimensional environmental impact, including ecosystem disruption, affecting nocturnal wildlife, altering plant growth, and contributing to skyglow [1,2,4,6]. Moreover, exposure to exposure to nighttime light affects human health [2,3,7]. Exposure to nighttime light pollution is associated with circadian and endocrine rhythms disruption and causes sleep disturbances, affects sleep quality, increases stress levels, affects metabolic functions as well as increases the risk of cardiovascular diseases [1–4,7]. Moreover, exposure to nighttime light pollution may increase the risk of some hormone-dependent cancers like breast or prostate cancer [7,8]. Night shift working is recognized by the International Agency for Research on Cancer (IARC) as a probable human carcinogen (group 2A) [9]. Some papers also linked exposure to nighttime light pollution with increased risk of obesity and mental disorders [10,11].

Exposure to light at night is also associated with hyperuricemia [12].

Prevention of environmental hazards is 1 of the individual interventions that may be implemented as a part of a healthy lifestyle. Most public health policies and environmental health interventions are focused on air pollution and noise pollution [13,14]. However, there is a lack of representative data on light pollution prevention measures implemented at the individual level. Personal mitigation strategies include limiting exposure to nighttime light, including the use of blackout curtains in the bedroom, limiting exposure to blue light, and changing lighting (from harsh and intense light to warm colors) [1–3]. In Poland, issues related to nighttime light pollution are underrecognized in public health policy and there were no nationwide campaigns on individual interventions to reduce nighttime light exposure.

Therefore, this study aimed to characterize individual interventions to reduce nighttime light exposure among adults in Poland as well as to identify factors associated with the implementation of individual interventions to reduce nighttime light exposure.

## MATERIAL AND METHODS

### Study design

This study constitutes a secondary analysis of data obtained from the nationwide cross-sectional survey entitled *Health Prevention and Health Inequalities* [15], conducted in December 2024 by the National Centre for Health Policy and Health Inequalities at Cardinal Stefan Wyszyński University, Warsaw, Poland. The original dataset was generated under a contractual agreement with the Polish Ministry of Education and Science (Agreement No. MEiN/2023/DPI/2717, dated October 13, 2023) [15]. The original dataset was made accessible under a policy permitting free-of-charge data use for scientific research purposes upon formal request to the Centre [15]. Raw datasets were acquired and analyzed following secondary analysis standards. A similar methodological approach was used in a previously published study [16].

### Population

The dataset was derived from a cross-sectional survey targeting a nationally representative sample of Polish adults aged 18–64 years [15,16]. Data collection was executed by ARC Rynek i Opinia [17], a professional public opinion polling company, on behalf of the National Centre for Health Policy and Health Inequalities [15]. The survey was administered online via a dedicated IT system. The sampling framework included stratification by gender, age group, educational attainment, and size of place of residence. National demographic statistics, as published by Statistics Poland, were utilized to address the sample's demographic structure [18].

### Measures

Individual interventions to reduce nighttime light exposure were assessed using the following survey item: “Which of the following interventions to reduce nighttime light exposure do you perform?” Available response options included: “I use blackout curtains in the bedroom,” “I turn off the indicator lights on electronic devices,” “I limit the screen time in the evening,” “I replace light bulbs with ones with a warmer color,” “none of the above.” Each item had dichotomous response options (yes/no).

Moreover, for the purposes of this analysis, 8 questions on personal characteristics were used.

### Ethics

This secondary data analysis followed the guidelines of the Helsinki Declaration. Participation in the study was voluntary and anonymous, with informed consent obtained from all respondents during the data collection process. The study protocol received ethical approval from the Bioethics Committee at the Medical University of Warsaw (Approval No. AKBE/56/2025).

### Statistics

Statistical analyses were conducted using IBM SPSS Statistics v. 29.0 (IBM Corp., Armonk, NY, USA). Sampling weights were applied to ensure demographic representativeness. Descriptive statistics were calculated and presented as frequencies and percentages. The χ^2^ test was used to assess associations between categorical variables. A multivariable logistic regression model was developed to identify factors associated with the adoption of ≥1 individual intervention to reduce nighttime light exposure. The 5 intervention-related items were aggregated into a single binary outcome variable. As there is a lack of scientific data on the factors associated with prevention of light pollution among adults in Poland, the authors decided to analyze factors associated with the implementation ≥1 individual intervention to reduce nighttime light exposure. Model fit was evaluated using Cox and Snell and Nagelkerke R^2^ statistics. Associations were reported as odds ratios (ORs) with 95% confidence intervals (CIs). A p-value of <0.05 was considered statistically significant.

## RESULTS

In the analyzed group (N = 5006), men constituted 50.1%. The age range of the respondents was 18–64 years. The mean age was 41.8 years (SD = 12.59 years) and the median was 42 years. Gender did not differ in the mean age (p = 0.163). Primary or vocational education was held by 31.8% of the respondents, secondary education by 37.9%, and higher education by 30.3%. Urban residents accounted for 59.4%, and rural residents for 40.6%. Over 75% of the respondents were employed (57.2% full-time and 18.0% part-time or casual). Detailed data are presented in Table 1.

**Table 1.** Characteristics of the study population based on the data from secondary analysis and original datasets generated in in the study among 5006 Poles aged 18–64 years, December 2024, Poland

Variable	Participants (N = 5006)	
	n	%
Gender		
male	2506	50.1
female	2500	49.9
Age		
18–24 years	541	10.8
25–34 years	990	19.8
35–44 years	1326	26.5
45–64 years	2149	42.9
Educational level		
primary or vocational	1593	31.8
secondary	1896	37.9
higher	1518	30.3
Place of residence		
rural area	2035	40.6
city		
<100 000 inhabitants	1581	31.6
100 000–499 000 inhabitants	789	15.8
≥500 000 inhabitants	601	12.0
Employment		
yes		
full-time	2862	57.2
part-time	902	18.0
no	1242	24.8
Self-assessment of financial situation		
“we have enough for everything and we're saving for the future”	999	19.9
“we have enough for everything without any special sacrifices, but we're not saving for the future”	977	19.5
“we live frugally and therefore have enough for everything”	1805	36.0
“we live very frugally to save for major purchases”	679	13.6
“we only have enough money for basic needs or we don't have enough money for even the cheapest food”	547	10.9

Among all respondents (N = 5006), the most common intervention to reduce nighttime light exposure was the use of blackout curtains in the bedroom (41.4% of respondents). Among females, those aged 18–24 years more often declared use of blackout curtains in the bedroom compared to those aged 45–64 years (49.5% vs. 37.6%, p < 0.001).

**Figure 1.**
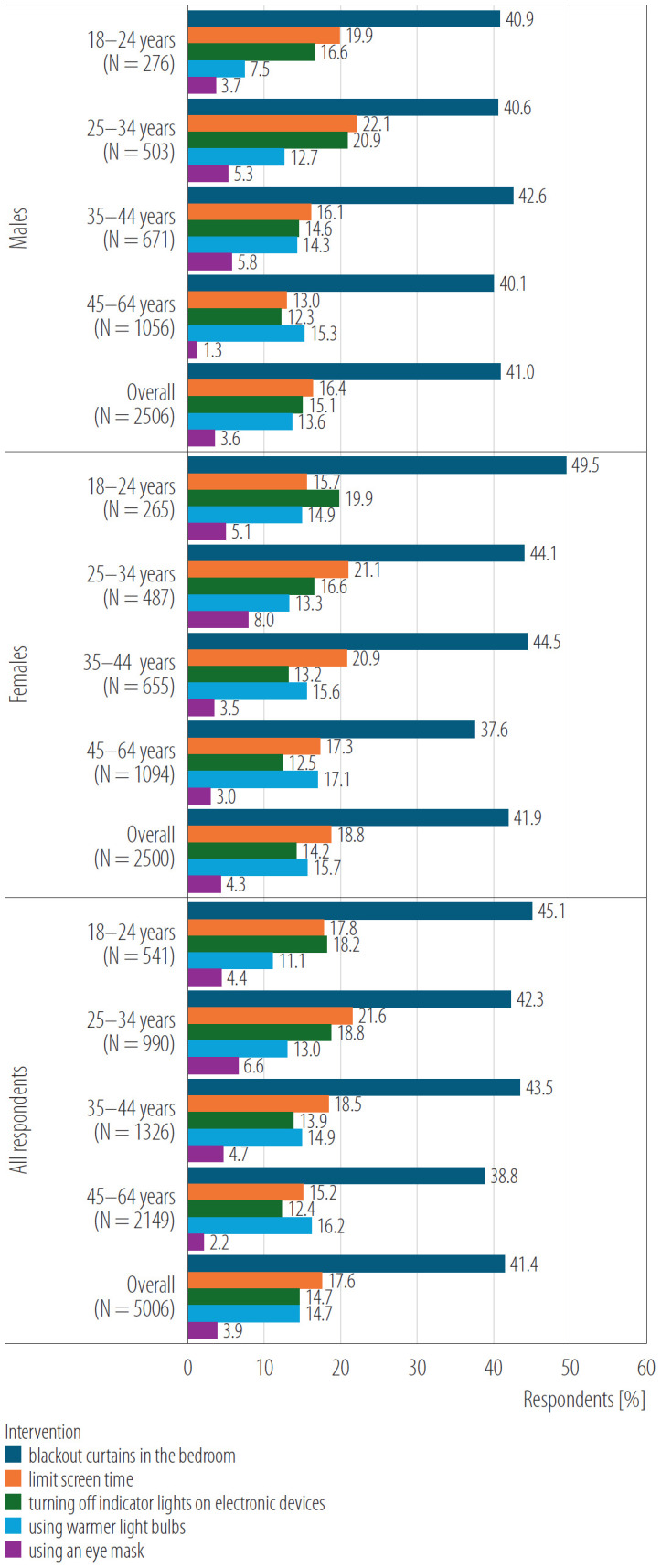
Individual interventions to reduce exposure to nighttime light pollution by age and gender in the study among 5006 Poles aged 18–64 years, December 2024, Poland

There were no significant age differences (p = 0.793) in the use of blackout curtains among males. The second most common intervention to reduce nighttime light exposure was limiting screen time in the evening (17.6% of respondents). Males aged 25–34 years (22.1%) were most likely to limit screen time in the evening, compared to those aged 45–64 years (13.0%, p < 0.005). There were no statistically significant differences in this form of individual intervention among females (p = 0.067). Another individual intervention to reduce nighttime light exposure was turning off indicator lights on electronic devices (14.7% of respondents) and using light bulbs with a warmer color (14.7% of respondents). The least common method was using an eye mask (3.9% of respondents). Detailed data are presented in Figure 1.

Among all respondents (N = 5006), 65% had implemented ≥1 individual intervention to reduce exposure to nighttime light pollution. The highest percentage was observed in the youngest age group, 18–24 years (71.4%), and the lowest in the oldest group, 45–64 years (60%). The highest percentage of respondents who implemented ≥1 individual intervention to reduce exposure to nighttime light pollution was in the group with higher education (68.2%), and the lowest in the group with primary or vocational education (59.4%, p < 0.001). People working full-time (65.7%) or part-time (72.8%) more often implemented interventions to reduce exposure to nighttime light pollution than those who were not working (57.4%, p < 0.001). Among the most affluent, 71.7% implemented ≥1 individual intervention to reduce exposure to nighttime light pollution, compared to the poorest groups where only 58% implemented individual interventions to reduce exposure (p < 0.001). Respondents who reported sleep problems occurring a few times a week (72.4%) more often implemented ≥1 individual intervention to reduce exposure to nighttime light pollution compared to those who did not experience such problems at all (56.3%, p < 0.001). However, gender (p = 0.147) and place of residence (p = 0.192) did not influence the obtained results. Detailed data, including specific methods of limiting exposure to nighttime light pollution, are presented in Table 2.

**Table 2.** Individual interventions to reduce exposure to nighttime light pollution by sociodemographic variables in the study among 5006 Poles aged 18–64 years, December 2024, Poland

Variable	Participants (N = 5005) [n]	Interventions [%]											
		blackout curtains in the bedroom (41.4%)	p	limiting screen time in the evening (17.6%)	p	turning off indicator lights on electronic devices (14.7%)	p	using warmer light bulbs (14.7%)	p	using an eye mask (3.9%)	p	at least 1 of 5 interventions (65.0%)	p
Gender			0.473		**<0.05**		0.402		**<0.05**		0.168		0.147
male	2506	41.0		16.4		15.1		13.6		3.6		64.0	
female	2500	41.9		18.8		14.2		15.7		4.3		66.0	
Age			**<0.01**		**<0.001**		**<0.001**		**<0.01**		**<0.001**		**<0.001**
18–24 years	541	45.1		17.8		18.2		11.1		4.4		71.4	
25–34 years	990	42.3		21.6		18.8		13.0		6.6		69.4	
35–44 years	1326	43.5		18.5		13.9		14.9		4.7		67.1	
45–64 years	2149	38.8		15.2		12.4		16.2		2.2		60.0	
Educational level			**<0.05**		**<0.001**		**<0.01**		**<0.001**		**<0.001**		**<0.001**
primary or vocational	1593	38.4		13.9		11.9		10.2		2.4		59.4	
secondary	1896	42.6		17.3		15.9		15.4		4.4		67.0	
higher	1518	43.2		21.9		16.1		18.5		4.9		68.2	
Place of residence			0.470		0.052		0.122		0.052		**<0.001**		0.192
rural area	2035	40.6		16.1		13.6		13.8		2.6		63.2	
city													
<100 000 inhabitants	1581	42.4		17.7		14.7		13.8		4.6		66.3	
100 000–499 000 inhabitants	789	40.4		19.3		15.3		16.7		5.3		66.0	
≥500 000 inhabitants	601	43.4		20.3		17.4		17.3		5.1		65.9	
Employment			**<0.001**		**<0.001**		**<0.001**		**0.004**		0.086		**<0.001**
yes													
full-time	2862	44.0		18.0		14.4		15.4		4.2		65.7	
part-time	902	38.0		23.4		20.7		16.3		4.5		72.8	
no	1242	38.0		12.3		10.9		11.8		2.9		57.4	
Self-assessment of financial situation			**<0.001**		**<0.01**		**<0.01**		0.148		**<0.05**		**<0.001**
“we have enough for everything and we're saving for the future”	604	48.1		21.3		18.1		16.4		4.0		71.7	
“we have enough for everything without any special sacrifices, but we're not saving for the future”	875	42.3		17.0		14.3		12.9		5.8		66.9	
“we live frugally and therefore have enough for everything”	1109	40.3		17.4		14.7		15.1		3.5		64.4	
“we live very frugally to save for major purchases”	789	38.3		16.8		13.4		15.0		2.8		59.3	
“we only have enough money for basic needs or we don't have enough money for even the cheapest food”	1629	35.5		13.7		10.8		12.8		3.4		58.0	
Sleep problems in the last 3 months			**<0.001**		**<0.001**		**<0.001**		**<0.001**		**<0.001**		**<0.001**
yes													
daily or almost daily	604	43.2		14.8		18.9		10.0		5.9		63.2	
a few times a week	875	44.8		18.9		18.3		12.4		7.4		72.4	
a few times a month	1109	46.3		18.3		15.5		16.5		3.9		70.8	
<1/month	789	39.7		21.9		14.8		16.8		3.0		67.8	
no	1629	36.5		15.3		10.5		15.4		1.8		56.3	
Looking for health-related information			**<0.001**		**<0.001**		**<0.001**		**<0.001**		**<0.001**		**<0.001**
no	1475	35.8		10.5		7.3		7.7		2.3		50.1	
yes	3531	43.8		20.6		17.8		17.6		4.6		71.2	

Bolded are statistically significant values.

A multivariable logistic regression model predicting implementation of ≥1 intervention to reduce exposure to nighttime light pollution yielded a Cox and Snell R^2^ of 0.068 and a Nagelkerke R^2^ of 0.094. Younger individuals were more likely to implement interventions to reduce exposure to nighttime light pollution. For those aged 18–24 years, the odds were 46% higher than for those aged 45–64 years (OR = 1.46, 95% CI: 1.17–1.82). Part-time workers were 62% more likely to implement ≥1 intervention to reduce exposure to nighttime light pollution (OR = 1.62, 95% CI: 1.33–1.96), compared to those who did not work. For those working full-time, these odds were 29% higher (OR = 1.29, 95% CI: 1.1–1.5). The wealthiest individuals were 60% more likely to implement intervention to reduce exposure to nighttime light pollution (OR = 1.6, 95% CI: 1.26–2.03) than those who lacked the means to even afford food. Those who reported sleep problems in the last 3 months were more likely to implement interventions to reduce exposure to nighttime light pollution. Those who experienced sleep problems daily or almost daily were 34% more likely to implement intervention to reduce exposure to nighttime light pollution (OR = 1.34, 95% CI: 1.09–1.64) than those who had no sleep problems. Respondents who experienced sleep problems several times a week had a 76% higher odds of implementing intervention to reduce exposure to nighttime light pollution (OR = 1.76, 95% CI: 1.46–2.12) compared to those who did not experience sleep problems. People who actively sought health information had a 117% higher odds of implementing intervention to reduce exposure to nighttime light pollution (OR = 2.17, 95% CI: 1.9–2.48) compared to those who did not seek health information. There were no significant associations (p > 0.05) between gender, educational level, and place of residence and the implementation of ≥1 intervention to reduce exposure to nighttime light pollution. Detailed data are presented in Table 3.

**Table 3.** Factors associated with the implementation of ≥1 individual intervention to reduce exposure to nighttime light pollution in the study among 5006 Poles aged 18–64 years, December 2024, Poland

Variable	p	OR (95% CI)
Gender		
male	0.835	0.99 (0.87–1.12)
female	ref.	ref.
Age		
18–24 years	**<0.01**	1.46 (1.17–1.82)
25–34 years	**<0.01**	1.29 (1.09–1.53)
35–44 years	**<0.05**	1.21 (1.04–1.41)
45–64 years	ref.	ref.
Educational level		
primary or vocational	ref.	ref.
secondary	0.267	1.09 (0.94–1.26)
higher	0.804	1.02 (0.86–1.22)
Place of residence		
rural area	ref.	ref.
city		
<100 000 inhabitants	0.162	1.11 (0.96–1.28)
100 000–499 000 inhabitants	0.969	1 (0.83–1.19)
≥500 000 inhabitants	0.675	0.96 (0.78–1.17)
Employment		
yes		
full-time	**<0.01**	1.29 (1.1–1.5)
part-time	**<0.001**	1.62 (1.33–1.96)
no	ref.	ref.
Self-assessment of financial situation		
“we have enough for everything and we're saving for the future”	**<0.001**	1.6 (1.26–2.03)
“we have enough for everything without any special sacrifices, but we're not saving for the future”	**<0.05**	1.31 (1.04–1.65)
“we live frugally and therefore have enough for everything”	0.267	1.13 (0.91–1.39)
“we live very frugally to save for major purchases”	0.404	0.9 (0.71–1.15)
“we only have enough money for basic needs or we don't have enough money for even the cheapest food”	ref.	ref.
Sleep problems in the last 3 months		
yes		
daily or almost daily	**<0.01**	1.34 (1.09–1.64)
a few times a week	**<0.001**	1.76 (1.46–2.12)
a few times a month	**<0.001**	1.69 (1.43–2.01)
<1/month	**<0.001**	1.45 (1.2–1.74)
no	ref.	ref.
Looking for health-related information		
no	ref.	ref.
yes	**<0.001**	2.17 (1.9–2.48)

Ref. – reference.

Bolded are statistically significant values.

## DISCUSSION

This study provides comprehensive data on individual interventions to reduce exposure to nighttime light pollution, implemented in a representative sample of working adults in Poland. Overall, 65% of adults aged 18–64 years had implemented ≥1 individual intervention to reduce exposure to nighttime light pollution. The use of blackout curtains in the bedroom was the most common intervention to reduce exposure to nighttime light pollution (41.4% of respondents). Age 18–44 years, having a full-time or part-time job, good financial situation, having sleep problems, and looking for health-related information were significantly associated (p < 0.05) with the implementation of ≥1 intervention to reduce exposure to nighttime light pollution. There was no significant impact (p > 0.05) of gender, educational level, and place of residence on the implementation of ≥1 intervention to reduce exposure to nighttime light pollution.

Prevention of environmental hazards is often omitted in public health policies in Poland, and most educational activities focus on air pollution prevention measures. In addition to population interventions aimed at reducing exposure to environmental hazards, personal interventions play a crucial role in preventing environmental hazards. Encouraging individuals to implement lifestyle behaviors that reduce exposure to environmental factors that can harm health is important [7]. Urban planning strategies aimed at reducing exposure to nighttime light in cities and dark-sky policies, including, among others, urban lighting management and rules for the use of neon signs and billboards, are basic population-based strategies to mitigate exposure to nighttime light pollution [7].

In this study, individual interventions to reduce nighttime light exposure were analyzed. Each of 5 interventions analyzed in this study protects against a specific source of light pollution. Using blackout curtains in the bedroom was the most prevalent intervention to reduce nighttime light exposure (41.4% of respondents). Blackout curtains may protect from artificial light from outdoor spaces, including traffic, streetlamps, and billboards. This intervention may be particularly important in highly urbanized areas [7]. However, in this study, there were no differences in the use of blackout curtains by place of residence.

Blue light from the screens is also a significant source of light pollution [4]. Nighttime smartphone use is associated with decreased sleep quality, lower sleep efficiency, high perceived stress, and severe depressive symptoms [19,20]. In the present study, only 17.6% of adults declared limiting screen time in the evening.

Multiple papers around the world raised the need to increase public health activities aimed at education on the role of limiting smartphone use and avoiding use of smartphones during bedtime [19–21]. Findings from the current study are in line with global scientific literature and also underline the need to implement educational campaigns on limiting smartphone use and other mobile devices before going to bed or during bedtime [20–22].

In the present study, 14.7% of respondents declared using warmer light bulbs or turning off indicator lights on electronic devices. Warmer light source indoors positively affects the production of the sleep hormone melatonin that have a positive impact on sleep [23]. Only 3.9% of respondents used an eye mask. This group may be the most exposed to nighttime light pollution, e.g., due to the housing conditions and living with other people (e.g., ≥2 people sleeping in 1 room, who have different habits and sleeping hours).

In multivariable logistic regression, age, having a full-time or part-time job, financial situation, having sleep problems, and looking for health-related information were significantly associated (p < 0.05) with the implementation of ≥1 intervention to reduce exposure to nighttime light pollution. Younger age groups are more likely to report implementing interventions to reduce nighttime light pollution. This is likely because the topic of light pollution is relatively new and information about it reaches audiences through new media, which younger generations use more often. The influence of age may indicate the significant role of social media in disseminating information on environmental hazards prevention methods. Working adults were more likely to implement interventions to reduce exposure to nighttime pollution that may result from the fact that they must pay special attention to the timing and length of their sleep due to the need to go to work. Good economic status is often associated with better health, including protection from environmental hazards [24]. Similarly, in this study, the wealthy groups were more likely to implement interventions to reduce nighttime light pollution compared to those with worse economic conditions. As expected, having sleep problems was associated with the implementation of interventions to reduce nighttime light pollution exposure. Those who experience sleep problems may seek methods that improve their quality of sleep, including limiting factors such as light pollution exposure that can disturb sleep. The strongest factor affecting the implementation of interventions to reduce light pollution in the model was seeking health information. It can be assumed that higher health literacy translates into preventive action. This observation requires further investigations and a separate study that measures health literacy levels and the implementation of preventive measures to limit environmental hazards.

There was no significant impact (p > 0.05) of gender, educational level, and place of residence on the implementation of ≥1 intervention to reduce exposure to nighttime light pollution. This observation suggests that demographic factors are less important when related to the prevention of environmental hazards, and other variables play a higher role.

### Practical implications

This study provides data on the prevention of environmental hazards – nighttime light pollution among adults in Poland. The level of implementation of individual interventions to reduce exposure to nighttime light pollution is relatively low, with blackout curtains as the most common interventions implemented in Poland. Findings from this study also show socio-demographic differences in the implementation of nighttime light pollution prevention measures among adults aged 18–64 years in Poland. Health information seeking was the most important variable associated with the implementation of ≥1 intervention to reduce exposure to nighttime light pollution. This observation underlines the role of health literacy in the prevention of environmental hazards and points out the need for further studies. This study underlines the need to implement nationwide public health campaigns on individual interventions to reduce exposure to nighttime light pollution, especially those simple measures that can be implemented with low or even no cost in households.

### Limitations

This study is based on secondary data analysis of an existing dataset obtained from a cross-sectional survey conducted via computer-assisted web interviews (CAWI), which constitutes a primary methodological limitation. The scope of the analysis was constrained by the variables available in the original dataset, which was specifically acquired for this research. Only 5 individual interventions to reduce exposure to nighttime light pollution were examined. Five individual interventions analyzed in this study do not exhaust the entire scope of possible actions aimed at reducing nighttime light pollution (e.g., wearing amber or orange blue-blocking glasses, use electronic devices with blue-light blocking filters). There were no specific measures of exposure in households. Outdoor and indoor exposures were not measured. However, even using satellite images and measuring light pollution would have been imprecise and would have added little to the study. Given the self-reported nature of the data, the potential for recall bias cannot be excluded. The CAWI method was used so households [18] that do not have Internet access (around 5% of households in Poland) were excluded from this study.

## CONCLUSIONS

There is a gap in the implementation of individual interventions to reduce exposure to nighttime light pollution among adults in Poland. Use of blackout curtains in the bedroom is the most common intervention, with low implementation of limiting screen time in the evening, which is an easy and non-cost intervention that can be widely implemented. Age, working status, financial status, having sleep problems, and attitudes towards health information seeking were significantly associated with the implementation of individual interventions to reduce exposure to nighttime light pollution. There is an urgent need to promote the prevention of environmental hazards among adults in Poland and to implement widespread education on how to recognize harmful environmental factors and respond when exposed to nighttime light pollution.
